# Supplementary material for: Optimal restoration for pollination services increases forest cover while doubling agricultural profits
Source: PLoS Biol. 2023 May 23;21(5):e3002107. doi: 10.1371/journal.pbio.3002107 (PMC10204975; doi:10.1371/journal.pbio.3002107)
Supplement: S1 Appendix — (DOCX) [file pbio.3002107.s001.docx]

**S1. Appendix for the article ‘*Optimal restoration for pollination services increases forest cover while doubling agricultural profits’***

# López-Cubillos et al. *Plos Biology*

## Methods

### *Appendix 2.1 | Data source for the study case*

We used the land cover maps from the Technological Institutional Repository of Costa Rica (1). The cover maps were produced by using satellite images obtained from USGS database (2005). These images were classified by trained analysts into seven categories: forest, non-forest, mangroves, clouds, cloud-produced shade, coffee crops and water (for more information about cover classification see Sánchez-Azofeifa et al. (2)). In this landscape, most non-crop vegetation is in remnant forest.

### *Appendix 2.2 | Coffee yield calculation, opportunity cost, and restoration cost*

To estimate yields according to bee foraging distances and abundance, we used the data reported by (3-8) for intact forest for Costa Rica (Table S2.1). With such abundances, we predicted the expected yield, using the distance decay function proposed by Lonsdorf et al. (5) and Ricketts (7, 8).

Table S2.1. Pollinator species, abundance, scaled abundance and foraging distance thresholds.

| Pollinator species | Averaged species abundances | Foraging distance threshold category (1 = 0-54; 2 = 55-87, 3=88-663) | Scaled abundance |
| --- | --- | --- | --- |
| Trigona (tetragonisca) angustula | 19.75 | 1 | 3.14 |
| Plebeia jatiformis | 51 | 1 | 8.10 |
| Plebia frontalis | 20.25 | 1 | 3.22 |
| Trigona (Tetragona) clavipes | 2 | 2 | 0.32 |
| Trigona dorsalis | 8.33 | 2 | 1.32 |
| Nannotrigona mellaria | 10.5 | 2 | 1.67 |
| Trigona fulviventris | 263.5 | 2 | 41.87 |
| Partamona cupira/Trigona fussipennis/Trigona corvina | 61 | 2 | 9.69 |
| Melipona fasciata | 18 | 3 | 2.86 |
| Apis mellifera | 187.33 | 3 | 29.77 |
| Trigonisca sp. | 38 | 3 | 6.04 |
| **TOTAL** | **679.66** |  | **108.00** |

To our knowledge, no study has measured bee abundance after restoration in the tropics. Therefore, based on Brosi et al. (9), where he found a direct relationship between plant diversity and bee abundance, we adapted the bee abundance from this study with plant species richness. At year zero from the restoration process, we used the bee abundance in pastures reported by Brosi et al. (9), and then we increased over the different time frames the bee abundance according to plant species richness reported by Aide et al. (10) in Costa Rica.

Table S2.2. Plant species richness in Costa Rica after restoration Aide et al. (11).

| Year | Plant richness | Percentage |
| --- | --- | --- |
| 5 | 8.21 | 35.65 |
| 10 | 11.76 | 48.83 |
| 20 | 17.17 | 71.28 |
| 30 | 20.87 | 86.65 |
| 40 | 23.02 | 100 |

Using the percentage values from table S2.2 (above), we estimated the potential bee abundance for restored forest by increasing the original bee abundance from pastures using the percentages until reach the scaled abundance reported by Lonsdorf et al. (5) (Table S2.3).

Table S2.3. Estimated bee abundance and scale abundance for restored forest.

| Year | Distances | Bee abundance | Scaled abundance |
| --- | --- | --- | --- |
| 0 | All | 60 | 9.534178854 |
| 5 | Shortest dist | 242.30 | 38.502 |
|  | Mid dist | 123.11 | 19.5628662 |
|  | High dist | 86.77 | 13.79 |
| 10 | Shortest dist | 331.88 | 52.7364 |
|  | Mid dist | 168.63 | 26.797194 |
|  | High dist | 118.85 | 18.89 |
| 20 | Shortest dist | 484.46 | 76.9824 |
|  | Mid dist | 246.15 | 39.113538 |
|  | High dist | 173.49 | 27.57 |
| 30 | Shortest dist | 588.93 | 93.582 |
|  | Mid dist | 299.24 | 47.547998 |
|  | High dist | 210.895833 | 33.510998 |
| 40 | Shortest dist | 679.66 | 108 |
|  | Mid dist | 345.335246 | 54.8748 |
|  | High dist | 243.386246 | 38.6748 |

Another important factor to consider for the calculation of yield is coffee bushes’ age. Coffee crops usually start producing yield after the second year of growth; however, yields are usually low until it reached five or six years when is its maximum production (12). Afterwards, from year seven or eight its production slowly decline; therefore, it is recommended to renovate the crops by pruning branches and also cutting the steam (Arcila et al. (12), p. 155). These actions can stabilize yields until it reaches 15 to 25 years, as production significantly decline, where it is recommended replanting (13, 14). We selected 20 years as the timeframe to consider full renovation. The average coffee production in Tarrazú between 2013-2014 and 2014 to 2015 was 215,308 fanegas. The total grown coffee area in 2014 was 4526 ha; therefore, they produced approximately 47.6 fan/ha (15). Coffee production cost in Costa Rican can be divided in high, mid and low producers (16). As coffee yields changes across plant maturity, we calculated the net present value considering three main groups of yields and time. For year 2 and 3 yields could reach a maximum of 29.9 fan/ha, for years 4 to 6 yields could reach a maximum of 44.3 fan/ha, from year 7 to 9 plants could produced the highest yields being 47.6 fan/ha. Then from year 10 to 14 yields were the same as years 4 to 6 and we assumed that from years 15 to 20 production decline similar to years 2 and 3. Finally, we assumed that after year 20, a total renovation would be needed. We only used coffee cost data from coffee fruit as we could not find data of the cost for drying coffee.

Restoration cost

We took the values reported by Sanchún et al. (17) (p.242) for the cost of a three-year restoration process in Costa Rica. As their values were reported to restore using linear tree planting, we adapted their values to an area of 40x40 m (cells size for our spatial planning framework, see below). To estimate the number of plants and materials for that size, we used values from Holl et al. (18) who estimated the number of plants needed for active restoration in plots of similar size (i.e. 50x50 m) in Costa Rica.

### Appendix 2.3| Coffee suitability analysis

We used eight biophysical variables to assess soil suitability for coffee crops in the same manner made described by López-Cubillos et al. (19) in their supplementary material.

**
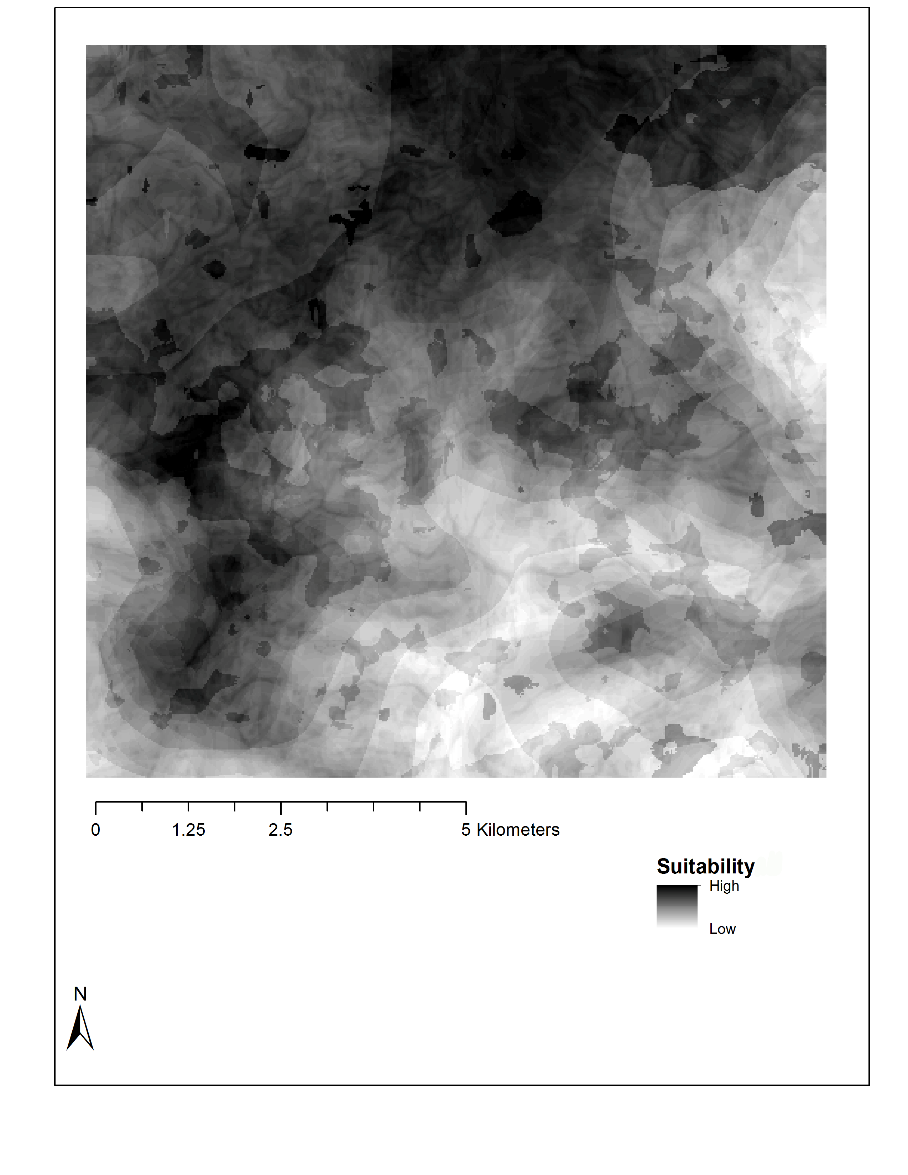
**

**Figure 2.1.** Land suitability for coffee crops in the case study area. The numerical data used for this map can be found in López-Cubillos et al. (19) **DOI** 10.1088/1748-9326/ac07d4 . The data underlying this Figure can be found in S6 Data.

### *Appendix 2.4 | Resutls*

Table S2.4 Sensitivity analysis results. The grey shading and bold characters represent the highest and lowest outcomes in the boxplot on figure six

| **Context** | **Sensitivity** | **Goal** | **NPV** | **Forest** | **Time_step** | **% Total forest change compared to baseline** | **% NPV change compared to baseline** |
| --- | --- | --- | --- | --- | --- | --- | --- |
| Only restoration | Original | Profit focus | 30110.73 | 147931.2 | 1 | 2.734259 | 137 |
| Only restoration | Original | Profit focus | 32718.03 | 148921.9 | 8 | 3.422292 | 158 |
| Only restoration | Original | Balanced | 25111.01 | 165918.1 | 1 | 15.22569 | 98 |
| Only restoration | Original | Balanced | 26566.6 | 170750.1 | 8 | 18.581419 | 109 |
| Only restoration | Original | Conservation focus | 6861.97 | 183329.9 | 1 | 27.317748 | -46 |
| Only restoration | Original | Conservation focus | -6959.15 | 201038.7 | 8 | 39.616035 | -155 |
| Only restoration | +25 forest intact | Profit focus | 35122.4 | 148604.2 | 1 | 3.20164 | 177 |
| Only restoration | +25 forest intact | Profit focus | 37753.67 | 149624.8 | 8 | 3.910407 | 197 |
| Only restoration | +25 forest intact | Balanced | 30790.03 | 163750.8 | 1 | 13.720567 | 142 |
| Only restoration | +25 forest intact | Balanced | 32407.24 | 168476.5 | 8 | 17.002444 | 155 |
| Only restoration | +25 forest intact | Conservation focus | 6861.97 | 183329.9 | 1 | 27.317748 | -46 |
| Only restoration | +25 forest intact | Conservation focus | -6959.15 | 201038.7 | 8 | 39.616035 | -155 |
| Only restoration | -25 forest intact | Profit focus | 27343.3 | 150384.5 | 1 | 4 | 115 |
| Only restoration | -25 forest intact | Profit focus | 30255.74 | 151613.9 | 8 | 5 | 138 |
| Only restoration | -25 forest intact | Balanced | 21567.11 | 168705.2 | 1 | 17 | 70 |
| Only restoration | -25 forest intact | Balanced | 22710.41 | 173382.6 | 8 | 20 | 79 |
| Only restoration | -25 forest intact | Conservation focus | 6861.97 | 183329.9 | 1 | 27 | -46 |
| Only restoration | -25 forest intact | Conservation focus | -6959.15 | 201038.7 | 8 | 40 | -155 |
| Only restoration | +25 forest restored | Profit focus | 34096.18 | 150216.3 | 1 | 4 | 168 |
| Only restoration | +25 forest restored | Profit focus | 38249.96 | 151571.4 | 8 | 5 | 201 |
| Only restoration | +25 forest restored | Balanced | 29534.52 | 164990.4 | 1 | 15 | 133 |
| Only restoration | +25 forest restored | Balanced | 33258.03 | 169402 | 8 | 18 | 162 |
| Only restoration | +25 forest restored | Conservation focus | 9236.5 | 183329.9 | 1 | 27 | -27 |
| Only restoration | +25 forest restored | Conservation focus | -6959.15 | 201038.7 | 8 | 40 | -155 |
| Only restoration | -25 forest restored | Profit focus | 27875.71 | 148528.8 | 1 | 3 | 119 |
| Only restoration | -25 forest restored | Profit focus | 29375.59 | 149488.6 | 8 | 4 | 131 |
| Only restoration | -25 forest restored | Balanced | 29534.52 | 164990.4 | 1 | 15 | 133 |
| Only restoration | -25 forest restored | Balanced | 33258.03 | 169402 | 8 | 18 | 162 |
| Only restoration | -25 forest restored | Conservation focus | 4487.45 | 183329.9 | 1 | 27 | -65 |
| Only restoration | -25 forest restored | Conservation focus | -6959.15 | 201038.7 | 8 | 40 | -155 |
| Only restoration | +25 restoration cost | Profit focus | 29980.57 | 147920 | 1 | 3 | 136 |
| Only restoration | +25 restoration cost | Profit focus | 32873.07 | 149133 | 8 | 4 | 159 |
| Only restoration | +25 restoration cost | Balanced | 24893.68 | 165381.6 | 1 | 15 | 96 |
| Only restoration | +25 restoration cost | Balanced | 26427.21 | 168779.1 | 8 | 17 | 108 |
| Only restoration | +25 restoration cost | Conservation focus | 5775.8 | 183329.9 | 1 | 27 | -55 |
| Only restoration | +25 restoration cost | Conservation focus | -8698.94 | 201038.7 | 8 | 40 | **-168** |
| Only restoration | -25 restoration cost | Profit focus | 30327.99 | 152882.8 | 1 | **6** | **139** |
| Only restoration | -25 restoration cost | Profit focus | 33024.11 | 154012.7 | 8 | 7 | 160 |
| Only restoration | -25 restoration cost | Balanced | 24785.91 | 167236.4 | 1 | 16 | 95 |
| Only restoration | -25 restoration cost | Balanced | 26057.87 | 172054 | 8 | 19 | 105 |
| Only restoration | -25 restoration cost | Conservation focus | 7948.14 | 183329.9 | 1 | 27 | -37 |
| Only restoration | -25 restoration cost | Conservation focus | -5219.36 | 201038.7 | 8 | 40 | -141 |
| Only restoration | +25 no pollination | Profit focus | 30542.6 | 147905.1 | 1 | 3 | 140 |
| Only restoration | +25 no pollination | Profit focus | 32744.05 | 148908.7 | 8 | 3 | 158 |
| Only restoration | +25 no pollination | Balanced | 25537.04 | 165918.1 | 1 | 15 | 101 |
| Only restoration | +25 no pollination | Balanced | 26566.56 | 170743.3 | 8 | 19 | 109 |
| Only restoration | +25 no pollination | Conservation focus | 7289.11 | 183329.9 | 1 | 27 | -43 |
| Only restoration | +25 no pollination | Conservation focus | -6959.15 | 201038.7 | 8 | 40 | -155 |
| Only restoration | -25 no pollination | Profit focus | 29684.7 | 147931.2 | 1 | 3 | 134 |
| Only restoration | -25 no pollination | Profit focus | 32737.02 | 148936.1 | 8 | 3 | 158 |
| Only restoration | -25 no pollination | Balanced | 24685.22 | 165919.6 | 1 | 15 | 94 |
| Only restoration | -25 no pollination | Balanced | 26564.88 | 170751.8 | 8 | 19 | 109 |
| Only restoration | -25 no pollination | Conservation focus | 6434.84 | 183329.9 | 1 | 27 | -49 |
| Only restoration | -25 no pollination | Conservation focus | -6959.15 | 201038.7 | 8 | 40 | -155 |
| Only restoration | 12% discount rate | Profit focus | 22032.26 | 147896.4 | 1 | 3 | **73** |
| Only restoration | 12% discount rate | Profit focus | 24088.76 | 149123.3 | 8 | 4 | **90** |
| Only restoration | 12% discount rate | Balanced | 15237.4 | 170653.2 | 1 | **19** | **20** |
| Only restoration | 12% discount rate | Balanced | 15505.27 | 176275.7 | 8 | 22 | **22** |
| Only restoration | 12% discount rate | Conservation focus | 3948.16 | 183329.9 | 1 | 27 | **-69** |
| Only restoration | 12% discount rate | Conservation focus | -6668.83 | 201038.7 | 8 | 40 | -153 |
| Only restoration | 6% discount rate | Profit focus | 43373.39 | 152935.1 | 1 | 6 | **241** |
| Only restoration | 6% discount rate | Profit focus | 47906.37 | 154322.6 | 8 | **7** | **277** |
| Only restoration | 6% discount rate | Balanced | 38542.18 | 163310.5 | 1 | **13** | **203** |
| Only restoration | 6% discount rate | Balanced | 42638.49 | 166661.7 | 8 | 16 | **236** |
| Only restoration | 6% discount rate | Conservation focus | 11728.61 | 183329.9 | 1 | 27 | **-8** |
| Only restoration | 6% discount rate | Conservation focus | -7274.15 | 201038.7 | 8 | 40 | -157 |
| Only restoration | 10% restoration failure | Profit focus | 30110.73 | 147931.2 | 1 | 3 | 137 |
| Only restoration | 10% restoration failure | Profit focus | 32986.28 | 147589 | 8 | 2 | 160 |
| Only restoration | 10% restoration failure | Balanced | 25111.01 | 165918.1 | 1 | 15 | 98 |
| Only restoration | 10% restoration failure | Balanced | 28142.62 | 162977.2 | 8 | 13 | 122 |
| Only restoration | 10% restoration failure | Conservation focus | 6861.97 | 183329.9 | 1 | 27 | -46 |
| Only restoration | 10% restoration failure | Conservation focus | -6013.08 | 187855.9 | 8 | 30 | -147 |
| Only restoration | 30% restoration failure | Profit focus | 30110.73 | 147931.2 | 1 | 3 | 137 |
| Only restoration | 30% restoration failure | Profit focus | 32945.63 | 146223.6 | 8 | 2 | 159 |
| Only restoration | 30% restoration failure | Balanced | 25111.01 | 165918.1 | 1 | 15 | 98 |
| Only restoration | 30% restoration failure | Balanced | 28484.33 | 157811.7 | 8 | 10 | 124 |
| Only restoration | 30% restoration failure | Conservation focus | 6861.97 | 183329.9 | 1 | 27 | -46 |
| Only restoration | 30% restoration failure | Conservation focus | -5012.13 | 177891 | 8 | 24 | -139 |
| Only restoration | 50% restoration failure | Profit focus | 30110.73 | 147931.2 | 1 | 3 | 137 |
| Only restoration | 50% restoration failure | Profit focus | 32582.63 | 145832.3 | 8 | 1 | 157 |
| Only restoration | 50% restoration failure | Balanced | 25111.01 | 165918.1 | 1 | 15 | 98 |
| Only restoration | 50% restoration failure | Balanced | 28104.02 | 156757.9 | 8 | **9** | 121 |
| Only restoration | 50% restoration failure | Conservation focus | 6861.97 | 183329.9 | 1 | 27 | -46 |
| Only restoration | 50% restoration failure | Conservation focus | -4470.76 | 174983.3 | 8 | **22** | **-135** |
| Expansion and restoration | Original | Profit focus | 41474.02 | 67399.58 | 1 | -53.192783 | 226.54137 |
| Expansion and restoration | Original | Profit focus | 44383.54 | 138838.6 | 8 | -3.580321 | 249.44921 |
| Expansion and restoration | Original | Balanced | 22206.03 | 169878.7 | 1 | 17.976228 | 74.83684 |
| Expansion and restoration | Original | Balanced | 33722.12 | 169226.4 | 8 | 17.523235 | 165.50757 |
| Expansion and restoration | Original | Conservation focus | 6861.972 | 183329.9 | 1 | 27.317748 | -45.97298 |
| Expansion and restoration | Original | Conservation focus | -6959.15 | 201038.7 | 8 | 39.616035 | -154.79216 |
| Expansion and restoration | +25 forest intact | Profit focus | 49330.92 | 68002.52 | 1 | -52.774062 | 288.40182 |
| Expansion and restoration | +25 forest intact | Profit focus | 52261.53 | 139546.6 | 8 | -3.088584 | 311.47568 |
| Expansion and restoration | +25 forest intact | Balanced | 29279.38 | 165755.2 | 1 | 15.112583 | 130.52813 |
| Expansion and restoration | +25 forest intact | Balanced | 42666.86 | 165094.7 | 8 | 14.653895 | 235.93308 |
| Expansion and restoration | +25 forest intact | Conservation focus | 6861.972 | 183329.9 | 1 | 27.317748 | -45.97298 |
| Expansion and restoration | +25 forest intact | Conservation focus | -6959.15 | 201038.7 | 8 | 39.616035 | -154.79216 |
| Expansion and restoration | -25 forest intact | Profit focus | 35958.26 | 70090.73 | 1 | -51.323852 | 183.11362 |
| Expansion and restoration | -25 forest intact | Profit focus | 38550.51 | 141138.6 | 8 | -1.983024 | 203.52338 |
| Expansion and restoration | -25 forest intact | Balanced | 19950.66 | 171195.5 | 1 | 18.890724 | 57.07947 |
| Expansion and restoration | -25 forest intact | Balanced | 29637.74 | 170666.3 | 8 | 18.523218 | 133.34967 |
| Expansion and restoration | -25 forest intact | Conservation focus | 6861.972 | 183329.9 | 1 | 27.317748 | -45.97298 |
| Expansion and restoration | -25 forest intact | Conservation focus | -6959.15 | 201038.7 | 8 | 39.616035 | -154.79216 |
| Expansion and restoration | +25 forest restored | Profit focus | 45452.38 | 69913.95 | 1 | -51.446622 | 257.86454 |
| Expansion and restoration | +25 forest restored | Profit focus | 49160.13 | 141109.8 | 8 | -2.002979 | 287.05717 |
| Expansion and restoration | +25 forest restored | Balanced | 27596.54 | 168131.2 | 1 | 16.762624 | 117.27851 |
| Expansion and restoration | +25 forest restored | Balanced | 40901.85 | 167409.6 | 8 | 16.261499 | 222.03646 |
| Expansion and restoration | +25 forest restored | Conservation focus | 9236.498 | 183329.9 | 1 | 27.317748 | -27.27739 |
| Expansion and restoration | +25 forest restored | Conservation focus | -6959.15 | 201038.7 | 8 | 39.616035 | -154.79216 |
| Expansion and restoration | -25 forest restored | Profit focus | 39234.89 | 68011.46 | 1 | -52.76785 | 208.91184 |
| Expansion and restoration | -25 forest restored | Profit focus | 40937.81 | 139362.7 | 8 | -3.21632 | 222.31958 |
| Expansion and restoration | -25 forest restored | Balanced | 19829.14 | 169834 | 1 | 17.94521 | 56.12269 |
| Expansion and restoration | -25 forest restored | Balanced | 28752.77 | 171608.3 | 8 | 19.17742 | 126.38191 |
| Expansion and restoration | -25 forest restored | Conservation focus | 4487.445 | 183329.9 | 1 | 27.31775 | -64.66857 |
| Expansion and restoration | -25 forest restored | Conservation focus | -6959.15 | 201038.7 | 8 | 39.61603 | -154.79216 |
| Expansion and restoration | +25 restoration cost | Profit focus | 41321.19 | 67426.95 | 1 | -53 | 225 |
| Expansion and restoration | +25 restoration cost | Profit focus | 44188.68 | 138787.1 | 8 | -4 | 248 |
| Expansion and restoration | +25 restoration cost | Balanced | 21930.4 | 169382.6 | 1 | 18 | 73 |
| Expansion and restoration | +25 restoration cost | Balanced | 33346.13 | 168517 | 8 | 17 | 163 |
| Expansion and restoration | +25 restoration cost | Conservation focus | 5775.8 | 183329.9 | 1 | 27 | -55 |
| Expansion and restoration | +25 restoration cost | Conservation focus | -8698.94 | 201038.7 | 8 | 40 | **-168** |
| Expansion and restoration | -25 restoration cost | Profit focus | 41670.04 | 72354.57 | 1 | -50 | 228 |
| Expansion and restoration | -25 restoration cost | Profit focus | 44680.13 | 143955.9 | 8 | 0 | 252 |
| Expansion and restoration | -25 restoration cost | Balanced | 22584.14 | 170293.7 | 1 | 18 | 78 |
| Expansion and restoration | -25 restoration cost | Balanced | 33911.43 | 170433.1 | 8 | 18 | 167 |
| Expansion and restoration | -25 restoration cost | Conservation focus | 7948.14 | 183329.9 | 1 | 27 | -37 |
| Expansion and restoration | -25 restoration cost | Conservation focus | -5219.36 | 201038.7 | 8 | 40 | -141 |
| Expansion and restoration | +25 no pollination | Profit focus | 41890.8 | 67375.57 | 1 | -53 | 230 |
| Expansion and restoration | +25 no pollination | Profit focus | 44370.27 | 138837.7 | 8 | -4 | 249 |
| Expansion and restoration | +25 no pollination | Balanced | 22617.67 | 169881.8 | 1 | 18 | 78 |
| Expansion and restoration | +25 no pollination | Balanced | 33450.09 | 169228.8 | 8 | 18 | 163 |
| Expansion and restoration | +25 no pollination | Conservation focus | 7289.11 | 183329.9 | 1 | 27 | -43 |
| Expansion and restoration | +25 no pollination | Conservation focus | -6959.15 | 201038.7 | 8 | 40 | -155 |
| Expansion and restoration | -25 no pollination | Profit focus | 41031.26 | 67428.45 | 1 | -53 | 223 |
| Expansion and restoration | -25 no pollination | Profit focus | 44003.35 | 138845.3 | 8 | -4 | 246 |
| Expansion and restoration | -25 no pollination | Balanced | 21783.56 | 169878.3 | 1 | 18 | 72 |
| Expansion and restoration | -25 no pollination | Balanced | 33824.45 | 169239.3 | 8 | 18 | 166 |
| Expansion and restoration | -25 no pollination | Conservation focus | 6434.84 | 183329.9 | 1 | 27 | -49 |
| Expansion and restoration | -25 no pollination | Conservation focus | -6959.15 | 201038.7 | 8 | 40 | -155 |
| Expansion and restoration | 12% discount rate | Profit focus | 30383.1 | 68126.51 | 1 | -53 | **139** |
| Expansion and restoration | 12% discount rate | Profit focus | 32409.27 | 138765.9 | 8 | -4 | **155** |
| Expansion and restoration | 12% discount rate | Balanced | 11324.32 | 175846.2 | 1 | **22** | **-11** |
| Expansion and restoration | 12% discount rate | Balanced | 17393.52 | 179804.3 | 8 | **25** | **37** |
| Expansion and restoration | 12% discount rate | Conservation focus | 3948.16 | 183329.9 | 1 | 27 | **-69** |
| Expansion and restoration | 12% discount rate | Conservation focus | -6668.83 | 201038.7 | 8 | 40 | -153 |
| Expansion and restoration | 6% discount rate | Profit focus | 59230.15 | 73269.79 | 1 | **-49** | **366** |
| Expansion and restoration | 6% discount rate | Profit focus | 63849.11 | 144004 | 8 | 0 | **403** |
| Expansion and restoration | 6% discount rate | Balanced | 38425.36 | 163865.5 | 1 | **14** | **203** |
| Expansion and restoration | 6% discount rate | Balanced | 56795.97 | 162019 | 8 | 13 | **347** |
| Expansion and restoration | 6% discount rate | Conservation focus | 11728.61 | 183329.9 | 1 | 27 | **-8** |
| Expansion and restoration | 6% discount rate | Conservation focus | -7274.15 | 201038.7 | 8 | 40 | -157 |
| Expansion and restoration | 10% restoration failure | Profit focus | 41474.02 | 67399.58 | 1 | -53 | 227 |
| Expansion and restoration | 10% restoration failure | Profit focus | 44361.48 | 137201.6 | 8 | -5 | 249 |
| Expansion and restoration | 10% restoration failure | Balanced | 22206.03 | 169878.7 | 1 | 18 | 75 |
| Expansion and restoration | 10% restoration failure | Balanced | 35806.54 | 160310.7 | 8 | 11 | 182 |
| Expansion and restoration | 10% restoration failure | Conservation focus | 6861.97 | 183329.9 | 1 | 27 | -46 |
| Expansion and restoration | 10% restoration failure | Conservation focus | -6326.51 | 187852.6 | 8 | 30 | -150 |
| Expansion and restoration | 30% restoration failure | Profit focus | 41474.02 | 67399.58 | 1 | -53 | 227 |
| Expansion and restoration | 30% restoration failure | Profit focus | 44206.25 | 135906 | 8 | -6 | 248 |
| Expansion and restoration | 30% restoration failure | Balanced | 22206.03 | 169878.7 | 1 | 18 | 75 |
| Expansion and restoration | 30% restoration failure | Balanced | 36384.36 | 154215.3 | 8 | 7 | 186 |
| Expansion and restoration | 30% restoration failure | Conservation focus | 6861.97 | 183329.9 | 1 | 27 | -46 |
| Expansion and restoration | 30% restoration failure | Conservation focus | -5352.82 | 177803.1 | 8 | 23 | -142 |
| Expansion and restoration | 50% restoration failure | Profit focus | 41474.02 | 67399.58 | 1 | -53 | 227 |
| Expansion and restoration | 50% restoration failure | Profit focus | 44097.72 | 135536.2 | 8 | -6 | 247 |
| Expansion and restoration | 50% restoration failure | Balanced | 22206.03 | 169878.7 | 1 | 18 | 75 |
| Expansion and restoration | 50% restoration failure | Balanced | 35984.77 | 153368.4 | 8 | **7** | 183 |
| Expansion and restoration | 50% restoration failure | Conservation focus | 6861.97 | 183329.9 | 1 | 27 | -46 |
| Expansion and restoration | 50% restoration failure | Conservation focus | -4634.95 | 174999.7 | 8 | **22** | **-136** |

**Reference**

1. Tec. Atlas de Costa Rica 2014. Costa Rica: Repositorio Institucional del Tecnológico de Costa Rica; 2014 2014.

2. Sánchez-Azofeifa A, Calvo-Alvarado J, Chong M, Castillo M, Jiménez V. Estudio de Monitoreo de Cobertura Forestal De Costa Rica 2005. Costa Rica: FONAFIFO, FundaTEC; 2007 2007.

3. Brosi BJ. The complex responses of social stingless bees (Apidae: Meliponini) to tropical deforestation. Forest Ecology and Management. 2009;258(9):1830-7.

4. Brosi BJ, Daily GC, Shih TM, Oviedo F, Durán G. The effects of forest fragmentation on bee communities in tropical countryside. Journal of Applied Ecology. 2008;45(3):773-83.

5. Lonsdorf E, Kremen C, Ricketts T, Winfree R, Williams N, Greenleaf S. Modelling pollination services across agricultural landscapes. Annals of Botany. 2009:mcp069.

6. Ngo HT, Gibbs J, Griswold T, Packer L. Evaluating bee (Hymenoptera: Apoidea) diversity using Malaise traps in coffee landscapes of Costa Rica. The Canadian Entomologist. 2013;145(4):435-53.

7. Ricketts T. Tropical Forest Fragments Enhance Pollinator Activity in Nearby Coffee Crops. Conservation Biology. 2004;18(5):1262-71.

8. Ricketts T, Daily GC, Ehrlich PR, Michener CD. Economic value of tropical forest to coffee production. Proc Natl Acad Sci USA. 2004;101(34):12579-82.

9. Brosi BJ, Daily GC, Ehrlich PR. Bee Community Shifts with Landscape Context in a Tropical Countryside. Ecological Applications. 2007;17(2):418-30.

10. Aide TM, Zimmerman JK, Pascarella JB, Rivera L, Marcano‐Vega H. Forest Regeneration in a Chronosequence of Tropical Abandoned Pastures: Implications for Restoration Ecology. Restor Ecol. 2018:328-38.

11. Verchot L, De Sy V, Romijn E, Herold M, Coppus R. Forest restoration Getting serious about the ‘plus’ in REDD+. Transforming REDD+ lessons and new directions. Bogor, Indonesia: CIFOR; 2018. p. 303.

12. Arcila P, Farfán V, Moreno B, Salazar G, Hincapié G. Sistemas de producción de café en Colombia. Chinchiná: Cenicafé; 2007 2007.

13. Peerdeman MA. Eficiencia relativa de pequeños y medianos productores de café en Comayagua Honduras 1991. Universiteit Amsterdam; 1991.

14. Thang TC, Burton MP, Brennan DC. Optimal replanting and cutting rule for coffee farmers in Vietnam. Cairns, Australia ed: Proceedings of the Australian Agricultural and Resource Economics Society (AARES) Annual Conference; 2009 2009.

15. Icafe. Informe sobre la actividad cafetalera de Costa Rica. San José, Costa Rica: Instituto del Café de Costa Rica; 2014-2015.

16. Icafe, Ueem. Costos de Producción Agrícola de Café Fruta Cosecha 2017-2018 - Fincas de 34 a 40 Und.400 L/ha; Fincas de 26 a 34 Und.400 L/ha; Fincas de 20 a 26 Und.400 L/ha. Instituto de Café de Costa Rica, Unidad de Estudios Económicos y Mercado; 2018.

17. Sanchún A, Botero R, Morera A, Obando G, Russo R, Scholz C, et al. Restauración funcional del paisaje rural: manual de técnicas. International Union for Conservation of Nature; 2016.

18. Holl KD, Zahawi RA, Cole RJ, Ostertag R, Cordell S. Planting Seedlings in Tree Islands Versus Plantations as a Large-Scale Tropical Forest Restoration Strategy. Restor Ecol. 2011;19(4):470-9.

19. López-Cubillos S, Runting RK, Mayfield MM, McDonald-Madden E. Catalytic potential of pollination services to reconcile conservation and agricultural production: a spatial optimization framework. Environ Res Lett. 2021.
