# Supplementary material for: Optimal restoration for pollination services increases forest cover while doubling agricultural profits
Source: PLoS Biol. 2023 May 23;21(5):e3002107. doi: 10.1371/journal.pbio.3002107 (PMC10204975; doi:10.1371/journal.pbio.3002107)
Supplement: Attachment — Submitted filename: Response_reviewers_v3.docx [file pbio.3002107.s008.docx]

| ID | Reviewer | Comment | Response |
| --- | --- | --- | --- |
| 1 | 1 | There is a lot to like in the paper.  In brief the authors present a framework to identify the best places for restoration in coffee plantations over a 40-year period following restoration.  They find that their restoration treatment increases forest area by 18% while doubling farmer profits after 40 years.   It's an interesting question to ask, using an interesting approach too, especially with regard to the spatial arrangement of the forest patches.  As a non-modelling field ecologist most of the parameters that went into the models made intuitive sense. I have a couple of major issues (which may just be the MS not being clear enough for the non specialist reader) and a few minor issues. | We are glad that the reviewer considers the framework we presented as relevant in the field and specially that they consider the parameters used in the model to be intuitive for field ecologists. Below we address the concerns that the reviewer has raised about the paper. |
| 2 | 1 | Line 117 states "It is expected that bee abundances will slowly recover after restoration until they are equivalent or closely equivalent to numbers observed in mature forest".   I'm not convinced that bee numbers will ever restore to that present in a mature forest - as it's not clear at this point how big these restored area are?  Forest area will likely affect how many species they have, as will how close patches of forest are and potentially their shape too (due to edge effects). I'm left wondering if bee abundance in a patchy and fragmented forest (Fig 3) ever be the same as that in mature forest (which by definition sounds a contiguous habitat rather than a patchy one)?   It doesn't need to be, it just needs to be considerably better than what was there before, which is a realistic outcome. | The reviewer expressed concerns about whether bee abundances will ever be fully recovered in the new restored forest patches. They posed the question about the size of the area restored. In this case, the area restored in the smallest patches is 1600 m2 (40 x 40 m), representing the grid cells as described in lines 132-134 in the Methods.  We agree that there is no evidence from the literature to suggest that such patches could recover completely or not, but there is however strong evidence of continued increases in abundance and diversity towards reference levels in similar landscapes. We consider that having restored patches that are small can significantly enhance pollinators’ populations as there is evidence in the literature showing that restoring with hedgerows (which are smaller than the smallest restoration patch from our study) can improve pollinator abundances in the short and mid-term (from one to ten years old (M'Gonigle et al., 2015; Morandin & Kremen, 2013; Ponisio et al., 2016)). We added a couple of sentence to make this point clear in the methods as follows:  Lines 179-185: “For instance, connecting crops to natural areas with hedgerows have had positive results for wild bee abundance and diversity. For example, after a year of growing hedgerows in farmland, M'Gonigle et al. (53) found bee abundance to have increased in size by 8%. Seven years later, they also found that species richness had increased compared to sites with no hedgerows. With mature hedgerows that are more than 10 years old, bee abundance was significantly higher when there was natural vegetation near crops (at least 100 m), compared to control sites (54)”  Lines 193-196 (new text underlined):  “To estimate bee diversity in ‘restored forest’ we assumed a direct relationship between plant richness and bee abundance, as reported by Kremen et al. (58) after a restoration action in Central Valley of California. For this, we used the plant richness reported by Aide et al. (59) and bee abundance as reported by Brosi et al. (60) in Costa Rica (Table S2.2 and Table S2.3). Therefore, bee abundances (Table S2.3) and pollination services increased gradually, as this depends on the distance from forest and the time since restoration commenced (Appendix S1-2.2). We also assumed that bee richness, abundance and pollination services would be completely restored after 40 years.”  Garratt et al. (2017) found a similar trend as we did with hedgerow restoration, as they found that in order to support pollinators’ abundance, a range of hedgerows and seminatural habitats are needed. In the results we found that we can maintain remanent forest and at the same time restore. We added to the discussion the following sentence:  Line 398-400: “Positive results for bees’ population recovery after small scale restoration approaches (such as hedgerow restoration) have been found in intensive agricultural landscapes after seven to ten years of restoration (53, 54, 77).”  However, we need to acknowledge that there might be some contrasting results in the literature regarding the benefits of restored areas to pollinators. For instance, Gutiérrez-Chacón et al. (2020) found that 13 years were not long enough to restore bee communities in a riparian forest in Quindio (Colombia). They however highlighted that species’ composition similarity showed progress in recovery and maybe more time would be needed, in addition to better management actions, such as fencing to protect habitat from cattle. Williams et al. (2011) show that species richness is similar between restored and reference sites (both riparian sites) in California, although bee composition differs. They also highlighted that pollination function (of native plants, not cropland) was also restored along pollinator abundance and richness, although being less robust in restored areas. We added a new paragraph in the discussion section in lines 389--405:  “Our model assumes that bee abundance will fully recover after 40 years in tropical forest and that the pollination benefit from restoration will be achieved between six to fifteen years of being restored, as substantial pollination benefits are provided by common species, such as Apis mellifera (29). These timelines are estimates for our case study system and should not be used as general assumptions that can be applied to other systems. The literature about the benefits of restoration on bee abundance are mixed, with some studies aligning with our assumptions and others unaligned. It is much better to set these timelines according to the known understanding of each landscape and forest system to which our method is applied and even then, care should be taken to acknowledge that recovery times can and do vary extensively within and across systems. Positive results for bees’ population recovery after small scale restoration approaches (such as hedgerow restoration) have been found in intensive agricultural landscapes after seven to ten years of restoration (53, 54, 77). In restored sections of riparian forest, however, Gutiérrez-Chacón et al. (78) and Williams et al. (79) found that bee communities are not similar to reference riparian areas or forests. Gutiérrez-Chacón et al. (78) highlighted that species composition showed progress in recovery in this system and suggested more time (>13 years) would be needed in addition to better management actions, such as fencing to protect habitat from cattle, to see a return to a species composition similar to reference sites” |
| 3 | 1 | Line 185: Is a 40 year timescale at all realistic in a working agricultural habitat? That is an entire working life of a farmer and I can't see any enthusiasm from farmers for a scheme with a payback period of 40 years? Or a government incentive that would work over this time period either? While I can see that this is based on the time taken for tropical forests to recover, there are very likely to be positive changes on biodiversity well before the 40 years are up? On line 235, it does say something about 20 years, but see my comments on this section in my next point. In the discussion (line 264) the authors talk about predictions 50 years after restoration work by other authors, so maybe this timescale is the norm in this field, in which case the reader needs a bit more information on the timescales in the introduction. Otherwise, the paper risks reading like an interesting theoretical exercise, but not a solution that is of any practical use, as farmers simply can't wait 40-50 yrs for a return on investment (here land lost to production). Nor does it have much real relevance to solving the biodiversity crisis if we have to wait 40 years for an outcome.. | The reviewer considers that a 40 year timescale might not be appropriate enough for decision making either for farmers nor governments. We completely agree that this is a potential problem but wanted to use time frames that were consistent with what information is available in the literature rather than more desirable timeframes for which we have no supporting evidence.  We selected this timeframe according a literature reviewed made on tropical forest restoration; however, we rephrased a paragraph to acknowledge the different time frames for both pollination benefits or plant species richness and density as explained in lines 158-168 as follows: " The time required to reach restoration benefits may vary according to the objective assessed. For instance, restoring crop fields with small flowering patches can start showing benefits within only 10 years (11), whereas the benefits to increase other biodiversity features such as plant species richness and density may require longer periods (<30 years) (48-50). Therefore, selecting a timeframe that can reflect positive outcomes not only for pollination services is important to foster more sustainable practices. Accounting for the difference on the benefits from restoration, we used a 40-years’ time horizon to calculated the Net Present Value (NPV) to determine coffee profitability. This timeframe was based on past studies of rainforest recovery rates from the tropics (i.e. Puerto Rico, Brazil, and Costa Rica, (48-51)). These studies showed that plant species richness increased in abandoned or restored areas (48, 49, 51) and in some cases aboveground biomass recovering showed similar values to old grown forest (50)"  Our results shows that both forest and the Net Present Value increases rapidly during the first five years, and then slowly stabilize in the next years until it reaches a plateau after 20 years for most of the goals (Figure 5 B). To highlight the benefits in a shorter term, we added a couple of graphs in Figure 4 showing the increasing economic benefits for a 5-year after restoration time. We also added more information about the benefits on the short term (after five years of restoration) in the following lines 282-293 (underlined text):  “Overall, profits increased ~100% or more in both contexts for the balanced goal (Fig. 4A and C), with a higher NPV in the ‘expansion and restoration” context in most of the cases because of the conversion of some intact forest to coffee production. ‘Profit focused’ was clearly the most profitable goal in the long term (after 40 years of restoration, Fig. 4C), but it had the poorest outcomes for forest, with intact forest decreasing by ~60% for the ‘crop expansion and restoration’ context (Fig. 4D). However, in a short term (ager 5 years of restoration, Fig. 4A), a balanced goal in an ‘only restoration’ context turns to be more profitable than the ‘expansion and restoration’ context (98% Vs 74% respectively). However, even when profit was the only goal, some forest was still restored (~5% in both contexts both in the short and long term) (Fig. 4B and C), but this was primarily in smaller patches relative to the other goals (Fig. 3)”  Furthermore, as mentioned in comment ID 2, the study made by Gutiérrez-Chacón et al. (2020) in Colombia explains that 13 years of restoration is not long enough to enhance bee species richness compared to forest or riparian counterpart, but the authors highlight that the recovery of some forest-associated species has been produced in some restored patches that function as potential corridors that connect forest patches. |
| 4 | 1 | Line 235: Here it says that a plateaux for forest benefits and profit are reached after 20 years, but I had a hard time understanding "Figure 5, diamonds, triangles and some of the square symbols".   Is it possible to calculate a break-even point as done (using a much simpler approach) in Blaauw et al (ref 8 in MS)?   Or is this what figure 5 shows, and I just can't make it out? | As there is much information in Figure 5 as we are adding the results for both contexts and the three goals, we understand that it might be a lot to take. Therefore, we decided to separate both contexts to unpack the results for the different goals. We hope this modification make this graph easier to understand. |
| 5 | 1 | Line 50 The authors say that it's not clear if restoring the native flora next to blueberries is cost effective citing Benayas & Bullock 2012 - however Blaauw & Isaacs 2014 clearly shown that it is cost effective for blueberries and the restoration work can pay for itself remarkably quickly. The authors point this out on line 60, which looks like contradiction to the Benayas & Bullock refference. Similarly, Pyewell et al. have shown that taking land out of production can quickly become cost effective eg. Pyewell et al. 2016 http://dx.doi.org/10.1098/rspb.2015.1740 | We agree with the reviewer about the point of restoration as a cost-effective solution, as pointed out by Blaauw & Isaacs 2014 for blueberry production (as shown in line 51). However, we wanted to highlight that restoration can be commonly perceived as a competing action to agricultural production as described by some authors such as Benayas & Bullock 2012 and Latawiec et al. 2015. To make this contrast clearer we rephrased the paragraph and added Pyewell et al. 2016 reference (thanks for the suggestion! It’s reference 8 in the text below) as follows (lines 41-54, underlined text):  “To address the global biodiversity crisis, landscape restoration has gained increasing traction (1), leading to initiatives such as the ‘Bonn challenge’, which aims to restore 350 million hectares of land (2), and the United Nations’ declare 2020-2030 as the ‘Decade on Ecosystem Restoration’. At the same time, food demand is expected to increase by 50-70% by 2050 (3), potentially expanding agricultural land-use and threatening biodiversity (4) along with crucial ecosystem services (5). Therefore, restoration can be perceived as both as a competing activity with agricultural land use and crop production (6, 7), and as an action than can cost-effectively protect biodiversity and increase agricultural outcomes through the flow of ecosystem services critical to agriculture, such as pollination (8). Pollination is a significantly important service in agricultural landscapes as 75% of agricultural crops benefit from it (9). Studies have quantified that the restoration of native flora adjacent to some berry crops (10) resulted in yield increases of 5-6% (11, 12) and by 1.5 kg per tree for all mango cultivars growing near wild flowering areas (13). However, whether these increased yields can compensate for the costs of setting aside restored lands at a landscape scale remains unclear”. |
| 6 | 1 | Line 126: Why is coffee bush age important to bees? | To calculate coffee profits over a 40-year timeframe, we need to account not only for pollination services, but also for coffee bushes’ age and land suitability (explain in lines 106-108).  It is not that the bees care about the age of the coffee bushes but coffee bush productivity changes with age – reflective of the plants life history strategy and investment in reproduction through time. Yields increase or decrease according to coffee bush age which was explained more in depth in the appendix S1-2.2 in lines 41-47. Nevertheless, we included the following explanation in lines 200-203 in the main text to add clarity (underlined text): " Finally, age of the coffee is another physiological important factor to be considered when calculating yields, as coffee starts producing fruit from the second year of growth, reaching a peak between year five and six (see appendix S1-2.2)" |
| 7 | 1 | Line 140 "To solve this problem" it's not clear what problem is being solved? | We agree with the reviewer that it is not clear what we referred by ‘problem’; therefore, we deleted this text leaving the following text which makes sense on its own. |
| 8 | 2 | The manuscript is well written, and the spatial optimisation framework offers interesting insights into a highly topical issue: the role of agriculture in forest landscape restoration. The paper is well structured, the figures are adequate, and the presentation is clear. I find the findings interesting and overall, this is a relevant work that demonstrates that restoration does not necessarily have to be in competition with agricultural crop production. There are, however, several improvements that can be made, largely to enhance the overall rationale and ensure that the findings are fully supported by the methodology. | We are thankful for the positive comments provided by this reviewer. Below we address the concerns that the reviewer has raised about the paper. |
| 9 | 2 | Introduction The overall rationale of the introduction lacks detail and could benefit from a more comprehensive review of up-to-date literature. To strengthen the introduction, I recommend the authors expand more on the definition of restoration in the context of this study and the role of natural habitats in increasing crop pollination. I missed reference to the global assessment on the value of biotic pollination and dense forest for fruit set of Arabica coffee by Moreaux et al. 2022 and a more detailed explanation of the role of wild pollinators in coffee production. Since there has been a lot of research on the dependency of coffee crops on pollinators, I would suggest including a more detailed overview of the current state of knowledge. Also, authors could include more detail about the context and different types of restoration, e.g. in L57f, e.g. a sentence about natural regeneration and active restoration (tree planting) and justify why the focus here is on active restoration. | The reviewer emphasises two points here. First, they mention the importance of adding more up-to-date and comprehensive literature regarding the importance of pollinators for coffee crops. Second they mention that we need to justify why we focused on active restoration compared to natural regeneration.  For the first point, we added more information not only about the importance of pollinators and coffee in general (we added three more papers regarding this topic), but also its importance on Costa Rican coffee as follows (lines 79-87):  “We used coffee as a case study given its importance as a cash crop in the Global South (23) and because it is known to benefit from pollination provided by wild bees (24-27). Indeed, while coffee can self-pollinate, the yields of *Coffee arabica* in particular can increase from 10-30% due to bee pollination (24). The specific system we used was coffee in Tarrazú, one of the main coffee producing canton (administrative division) in Costa Rica. Coffee production in this region has been shown to decrease by 18% when crops are too far from forest (28, 29). Using this system we answer the following questions: 1) To what extent can the strategic allocation of land for restoration achieve both conservation and crop production goals? 2) How do these benefits change over time? and 3) How important is agricultural expansion to these outcomes?”  For the second point, we focused on active restoration as we wanted to explore the potential to obtain greater pollination service benefits within sun coffee crops. The literature regarding natural regeneration nearby coffee plantations mentions that natural regeneration was possible where there was a decline in coffee production, so land was marginal for production (Chazdon & Guariguata, 2016). Thus, we think natural regeneration is not the best approach for our study as the idea is to offer a framework to farmers or stakeholders that are actively working in coffee crops and that are not planning to abandon them. Further, given the time-frame to receive benefits is already long (as noted by reviewer 1), modelling natural regeneration would further prolong this. In a long-term experiment (19 years) in Costa Rican coffee and cattle farms, Holl et al. (2020) found that active restoration applied through nucleation can attract greater numbers of birds, bats, epiphyte species, and tree recruitment compared to natural regeneration. Therefore, based on Holl et al. (2011) we estimated the number of plants needed for active restoration as explained in Appendix 2.2 in the ‘Restoration cost’ section. We do agree with the reviewer that more information regarding the type of restoration should be described in the main text to add more clarity, so we added to the methodology the following (lines 100-112):  “While natural regeneration can be a cost-effective option for biodiversity protection and the maintenance of ecosystem services, this action is usually most possible in lands of marginal production (33). For instance, in countries like Puerto Rico and Brazil, declining rural populations in coffee landscapes is opening the window for more natural regeneration as land is abandoned (34). However, the aim of this paper is to provide a framework for expansion of natural areas in landscapes used for active agricultural production, so we focused on active restoration, as it has been shown to provide effective outcomes in coffee regions in Costa Rica. For instance, Holl et al. (35) show that active nucleation (clusters of plants within a large area) can attract greater numbers of birds, bats, epiphyte species, and tree recruitment compared to natural regeneration in coffee and cattle lands after a decade of restoration. Therefore to model the costs of active restoration we estimated the number of plants and materials for a 50x50 m plot size using values from Holl et al. (36).” |
| 10 | 2 | Methods The spatial optimisation framework introduces an original way of assessing the benefits of strategic land management to crop production and forest conservation. However, the methodology needs to be clarified in several instances. For example, could the authors justify more clearly **why land suitability, pollination services and coffee bushes' age were included as the factors to estimate coffee yield, and how the factors were weighed?** Also, I think the differences between **the "agricultural context" and "goals" presented in Figure 1 need to be more clearly explained**. For example, what is the difference between the "only restoration" agricultural context and the "conservation focused" goal? Further, could the "agricultural context" be understood as different scenarios (e.g. similar to De Pinto et al. 2020, a global analysis of the effects of involving crop production in restoration efforts, who based their modelling approach on a comparison of a business-as-usual scenario with a series of alternative scenarios)? I would urge the authors to clarify their approach in the methods section. | The reviewer considers that we need to clarify to aspects:   1. Clarify the use of (i) land suitability, pollination services and coffee bushes' age for coffee yield estimations and the weights. Because much of the information regarding yield calculation is in the appendix material, we decided to move a substantial portion of it to the main text to add more clarity. We also reorganized part of the text from section ‘planning goals and optimization analysis’ to make things clearer.   To make this clearer, we make three main modifications:   A. Lines 206-233: We moved the first paragraph from the ‘Planning goals and optimization analysis’ to the ‘Coffee yield and profit calculation’ which described that we divided the landscape in small grids to assess the potential yield for each one of them. B. Afterwards, we moved some of the appendix material up to the ‘Coffee yield and profit calculation’ to describe the distance decay function proposed by Lonsdorf et al. (2009) and Ricketts (2004) , which explain the potential yield using values of bee abundance (Lines 145-157). We also explain here how we introduce coffee land suitability and coffee’s age into the equation (eq1). C. Finally, we also moved from the Net Present Value equation (eq2) and its respective description from the appendix to the same section to add more clarity (lines 169-176)      2. To clarify the differences in the framework between agricultural context and goals we:      A. We first added the following text in the first paragraph of the methods (underlined text in lines 89-98):   “In this paper, we present a novel optimization framework for the spatial and temporal allocation of restoration in agricultural landscapes (Fig. 1). The framework aims to maximize coffee profitability and forest restoration and retention in two different agricultural contexts. In the first context, there is no intention of expanding coffee production within the remanent forest but instead it only seeks to restore agricultural land (named as ‘Only restoration’ here after). In the second context, both restoration and agricultural expansion are explored (named as ‘Expansion and restoration’ here after). We also used a ‘Baseline’ context, which represents the current landscape without any coffee expansion nor restoration in agricultural lands.”   B. We also modified Figure 1 and show how goals are a product of each agricultural context, as they represent different scaled values on a pareto frontier curve, which represents the trade-offs between coffee profitability and forest. Figure 1 is in the separate document and at the end of this table. |
| 11 | 2 | Results and discussion On several occasions, the authors make statements about their findings that are not fully aligned with their research questions and method. I recommend the authors carefully revise the results and discussion to ensure that the reported findings are fully supported by the methodology. For example, L267f "[…] ecosystem services to agriculture, such as pollination, which we have shown are crucial in delivering economic benefits". **I suggest the authors rephrase this statement, as their results do not show the importance of pollination in delivering economic benefits, but rather use this as a critical assumption for the spatial optimisation framework**. In the abstract, **authors state that strategic restoration can increase forest cover by 18%, however this result is not mentioned in the results section (only in Table S2.4 under % total forest change compared to baseline). Is it possible to report this in the results section as well?** | The reviewer makes a couple of suggestions:   1. We want to highlight that our framework considers the economic benefits derived by pollination services through the assessment of the Net Present Value (NPV) explained in lines 169-176 and also in lines 30-54 in the appendix S1. It seems that our presentation of these methods were not as clear as they could have leading to confusion about the NPV. The NPV includes economic costs, and potential profits, both of which vary over space and time. Therefore, it is far from a foregone conclusion that pollination delivers (net) economic benefits in this landscape, as the amount, location, and timing of restoration can alter the economic costs and benefits (hence why we use spatial optimisation to find the best solutions). We have rephrased the text in the methods (lines 63-168) and hope now that with the new text has solved this issue. 2. The reviewer mentioned that we needed to highlight the significant positive outcomes from restoration. We now do so on lines 276-279 as follows: “The ‘balanced’ goal achieved substantial conservation and economic benefits. In this case, restored forest increased ~20% for both contexts compared to the baseline (both in the short and long term, Fig. 4B and D) and includes a mix of large and small restoration patches (Fig. 3)” |
| 13 | 2 | L26f: Rephrase this slightly, e.g. 75% of the global leading food crops, and add the reference in the main text (Klein, A., B. Vaissière, J. Cane, I. Steffan-Dewenter, S. Cunningham, C. Kremen, and T.Tscharntke. 2007. Importance of pollinators in changing landscapes for worldcrops.) | We thank the reviewer to make a suggestion to improve the readability of the sentence - we have rephrased as suggested. We have now added a sentence regarding the 75% benefit in the introduction in order to be able to add the reference (line 49): “Pollination is a significantly important service in agricultural landscapes as 75% of agricultural crops benefit from it (Klein et al., 2007).” |
| 14 | 2 | L31: from restored vegetation or generally from vegetation? Maybe the authors could put restored in brackets? | We agree with the reviewer, and we made the suggested modification. |
| 15 | 2 | L50 / L60f and L70f: Is the study by Blaauw and Isaacs (2014) the only one that looks at benefits of restoration to biodiversity and crop productivity? Would it be possible to expand a bit more on this and include other literature? | We agree with the reviewer, and we have added three more case studies to support how native flora can enhance pollination services in line 50 we added the following information:  “Studies have quantified that the restoration of native flora adjacent to some berry crops (10) resulted in yield increases of 5-6% (11, 12) and by 1.5 kg per tree for all mango cultivars growing near wild flowering areas (13).” |
| 16 | 2 | L32: determine | The word ‘determine’ is used on line 32 of the abstract, but we cannot find any grammatical issues with its use in that sentence. However, we have made edits throughout the manuscript for proper English usage. |
| 17 | 2 | L79: 3) How important is agricultural expansion to these outcomes? (Capital H) | We thank the reviewer for spotting the typo! |
| 18 | 2 | Examples of restoration benefits to crop yield: mention blueberries | As the reviewer did not specify the line on the text for this comment, we are assuming that they might be referring to lines 74 to 78, where we stated there is no study that considers the demand of pollination services, pollinator abundance in a dynamic temporal manner.  We think the reviewer is referring to the blueberry study made by Blaauw and Isaacs (2014) where they did account for bee abundance and the temporal dynamics; however, they did this at a farm scale, rather than a landscape scale. Thus, we made the following changes in those lines as follows (underlined text):  “However, no studies, to our knowledge, have simultaneously considered demand for pollination services at a landscape scale (rather than at a farm scale as done by Blaauw and Isaacs (11)), and the changes for pollinator abundance with the services they provide in a dynamic temporal manner over a specific timeframe, including the economic returns they deliver.” |
| 19 | 2 | L107f: rephrase slightly, for example "pollination services, land suitability and coffee bushes' age were selected as the factors to estimate coffee yield" Why only these ones, justify | We have significantly changed this paragraph based on previous comments. We have now justified why we used these three parameters and why we did not include farm management factors (lines 138’144):  “[…] For each cell, we identified the current land use type and calculated coffee yield and profitability. We used three commonly used parameters in the literature to assess coffee yields: coffee land suitability, coffee plant age, and the potential pollination services (29, 39-44). Other parameters such as farm management practices (the use of fertilizes, water, etc) could also be included in the assessment of coffee yields (45, 46); however, because farm practices can vary widely across the agricultural landscape and are difficult to ascertain, we decided to exclude these factors.” |
| 20 | 2 | L281: Although PES are a very interesting solution for the mismatch in temporal economic costs and benefits of restoration, it is not central to the findings of this study. Shorten the section or include it later on in the discussion, after the results of this study have been discussed in more detail and embedded in wider literature | We agree with the reviewer that the discussion of PES is located too early in the discussion, so we opted to move the entire section lower in the discussion, when we are starting to develop more general topics (lines 412-427). |
| 21 | 2 | L283: typo: types OF schemes | We thank the reviewer for the comments regarding grammar and we have made the suggested modification. |
| 22 | 2 | L300: not full sentence, rephrase | We added the following words to complete the sentence (underlined): “Our restoration solutions could feasibly involve multiple landholders in restoration efforts, some of which are likely to be smallholders (as is very common in Costa Rica).” |
| 23 | 2 | L342: some "level" of restoration ? | Yes, adding level in the sentence improves it. We thank the reviewer for the advice. |
| 24 | 2 | L531: not full sentence, rephrase | We thank the reviewer for highlighting this error. We rephrased the sentence as follows (line 18, new text underlined):  “Therefore, based on Brosi et al. (34), where he found a direct relationship between plant diversity and bee abundance, we adapted the bee abundance from this study with plant species richness” |
| 25 | 2 | L535: typo: Costa Rice (not Corsta Rica) | We thank the reviewer for spotting the typo! |

**Figure 1 for comment ID 10.** Spatial optimization framework. In the ‘*Agricultural context’* box, three different contexts are represented. The ‘Baseline’ context is the current landscape where there is no coffee expansion or restoration. In the ‘Only restoration’ context, restoration is allowed to happen within the coffee cropland, but no coffee expansion is allowed. In the ‘Expansion and restoration’ context, both coffee expansion in intact forest and coffee cropland restoration is allowed across the landscape. The ‘Model input’ box represents all the biophysical and economic data needed to run the optimization (see methods). In the ‘*Optimization*’ boxes we want to simultaneously maximize the coffee NPV and forest habitat for each agricultural context. The ‘*Sensitivity analysis*’ box, four sets of sensitivity analyses are conducted on key variables (see methods). Finally, the ‘*Pareto frontier’* box represents the trade-offs between maximizing the NPV or forest. In this box we can highlight three main goal for each optimization: 1. The ‘Conservation Focus Goal’ gives more priority to restoration, 2. the ‘Profit Focus Goal’ gives more priority to agricultural profit (net present value (NPV)), and 3. the ‘Balanced Goal’ aims to find an equilibrium between both objectives.

 **Figure 5.** Cumulative forest (A. and C.) and Profit (Net Present Value, NPV) index (B. and D.) over the time horizon (40 years) for the only restoration and expansion and restoration contexts. The data underlying this Figure can be found in S2 Appendix.
