# Supplementary material for: Optimal restoration for pollination services increases forest cover while doubling agricultural profits
Source: PLoS Biol. 2023 May 23;21(5):e3002107. doi: 10.1371/journal.pbio.3002107 (PMC10204975; doi:10.1371/journal.pbio.3002107)
Supplement: Attachment — Submitted filename: Response_minor_comments_v4.docx [file pbio.3002107.s009.docx]

Response to minor revision comments:

1. L33f: The authors have clarified the methods and results, which are now better aligned. However, the authors write in the abstract: "We present a novel planning framework to determine the best locations for restoration in agricultural landscapes while accounting for yield improvements over 40-years following restoration." From my understanding of the methods and results, this does not align fully with what is described in the manuscript, as the study did not aim to determine where the best locations are, but rather determine the best strategic allocation of restoration in agricultural landscapes (spatial arrangement rather than location). I would suggest the authors clarify this in the abstract.

- We changed the word ‘location’ to ‘spatial arrangement’ in line 33 as the reviewer suggested.

2. L51f: In response to my previous comment to reference the global assessment on the value of biotic pollination and dense forest for fruit set of Arabia coffee by Moreaux et al. 2022, the authors have referred to the work of Moreaux et al. 2022 in the added sentence "Indeed, while coffee can self-pollinate, the yields of Coffee arabica in particular can increase from 10-30% due to bee pollination (24)". However, this statement is not a finding of Moreaux et al.'s work, but a citation of other publications in Moreaux et al.'s article, see "Coffee is able to self-pollinate, but it significantly benefits from biotic pollination with reported fruit set increases of 10-30% in Coffea arabica L., as compared to self-pollination (Hipólito et al., 2018, Klein et al., 2003a, Saturni et al., 2016)." I recommend the authors clarify this and cite the original articles. Also, I suggest the authors have a closer look at the global assessment of the value of biotic pollination and dense forest for fruit set of Arabica coffee, as the findings of that study are very relevant to the rationale of this study.

- I have made the changes to the citations accordingly to the reviewer’s comment adding Bravo-Monroy et al., 2015 and Saturni et al., 2016, which represent the ranges of coffee yield variation written in the manuscript (from 10% to ~30%).
- Although the paper from Moreaux and collaborators (2022) does not address the importance of restoration, their paper is relevant our discussion on the importance of maintaining forest remnants, we added the following text in lines 375-382 (changes underlined):

“Caution is thus needed with crop expansion, as removing nature to increase economic gains will reduce the resiliency of the ecosystem and increase environmental variability (e.g., longer droughts) and potentially lead to ecosystem collapses if not managed in line with our understanding of the ecology of fragmented landscapes (73). Furthermore, some landscapes with dense forest cover (≥75% canopy) that are located nearby coffee crops (~1km) have higher pollination richness and fruit set due to pollination services (Moreaux et al., 2022); therefore, focusing only on restoration rather than deforestation and restoration at the same time would be the best strategy.”

3. Minor comment: short title has a typo (restoration)

- Thank you for spotting the typo! We made the corresponding correction.
